# Correlative ecological niche model applications to predicting landscape-scale woody plant encroachment in Kansas tallgrass prairie systems

**DOI:** 10.1371/journal.pone.0305168

**Published:** 2024-06-13

**Authors:** A. Townsend Peterson, Yuan Yao, Marlon E. Cobos, Xiangming Xiao

**Affiliations:** 1 Biodiversity Institute, University of Kansas, Lawrence, Kansas, United States of America; 2 School of Biological Sciences, Center for Earth Observation and Modeling, University of Oklahoma, Norman, OK, United States of America; Satyawati College, University of Delhi, INDIA

## Abstract

Woody plant encroachment (WPE) in grassland ecosystems has been a pervasive process across the Great Plains, yet a predictive understanding of where it will occur has been elusive. As an exploration of tools of potential utility in this challenge, we mapped WPE processes over the years 2015–2021 in a set of 9 counties in central Kansas. We developed and tested two correlative models based on landscape features: one that assessed distribution of evergreen trees in 2015, and another that assessed areas of WPE in succeeding years. Both models were successful, being able to predict 2015 forest distributions and being able to predict WPE during 2015–2021, as functions of characteristics of landscapes. These simple, correlative models will certainly not be able to predict WPE processes globally, or even regionally, but provide first proof-of-concept explorations for the central Great Plains region.

## Introduction

Woody plant encroachment (hereafter WPE) into grassland ecosystems is a process that has important consequences for ecosystem structure and function and for biodiversity [1–3]. WPE is a phenomenon that is also quite widespread—indeed in some regions pervasive—geographically, comprising a dominant vegetational across important swaths of the Earth’s surface. As such, understanding, characterizing, and anticipating the geographic course of WPE represents an important challenge for landscape ecology and biology.

WPE represents the coarse-scale manifestation of local population processes, particularly as regards certain invasive tree species: in tallgrass prairie systems, the most common tree species is the eastern red cedar (*Juniperus virginianus*). Specific biotic and abiotic drivers of WPE by red cedar remain topics of debate, although clearly fire suppression represents a crucial long-term driver promoting WPE [4]. It appears that plant species richness and diversity have no consistent association with red cedar encroachment, such that resource availability and/or other biotic or abiotic factors may be better predictors of WPE [5]. Human demographic changes appear also to promote WPE by red cedar [6]. The mechanisms promoting WPE may even go so far as to include allelopathic interactions, in which red cedars inhibit grass growth once the trees are able to establish [7].

Numerous studies have mapped WPE on various spatial extents [8,9], or have characterized WPE implications for ecosystem function [1–3], water resources [10] and climate [11]. Although several studies have sought to identify landscape-scale drivers of WPE [12,13], few have taken on the harder challenge of attempting to predict the spatial course of WPE processes. Nonetheless, such a predictive understanding of WPE processes would allow considerable advantage in mitigating negative aspects of WPE on diverse landscapes, such as grassland degradation, reduction of cattle production, and loss of water resources.

Here, we explore application of correlative techniques that have been developed in biodiversity science and ecology to characterize and predict the geographic potential of individual species to understanding WPE processes. These techniques take advantage of the so-called Hutchinsonian Duality: a dual, geographic and environmental space in which species are distributed [14–16]. We fit correlative models of environmental requirements of species, and use those models to estimate and anticipate their geographic potential. These models have been applied previously to understand distributions of species assemblages [17]—ecological communities, as it were—so they may have utility in anticipating WPE processes.

As such, the purpose of this paper is to explore the application of correlative ecological niche models to anticipating WPE. Specifically, we focus on eastern red cedar in the Flint Hills tallgrass prairie region of central-eastern Kansas, and the WPE process that centers on that species in a 9-county region of the Flint Hills. We use a new remote-sensing dataset developed by our group, comparing evergreen woody plant (i.e., red cedar, which is pretty much the only evergreen tree in the region) distributions between 2015 and 2021. In a first suite of modeling efforts, we develop predictive models for the distribution of the species across the study area in 2015. Subsequently, we use similar techniques to identify areas where WPE occurred between 2015 and 2021. We apply models developed across one Kansas county (Wabaunsee County) to the 8 surrounding counties, and find clear and excellent predictions in both cases, suggesting that landscape drivers can be identified that relate to both red cedar distributions (in 2015) and WPE by red cedar between 2015 and 2021.

## Methods

**Fig 1:**
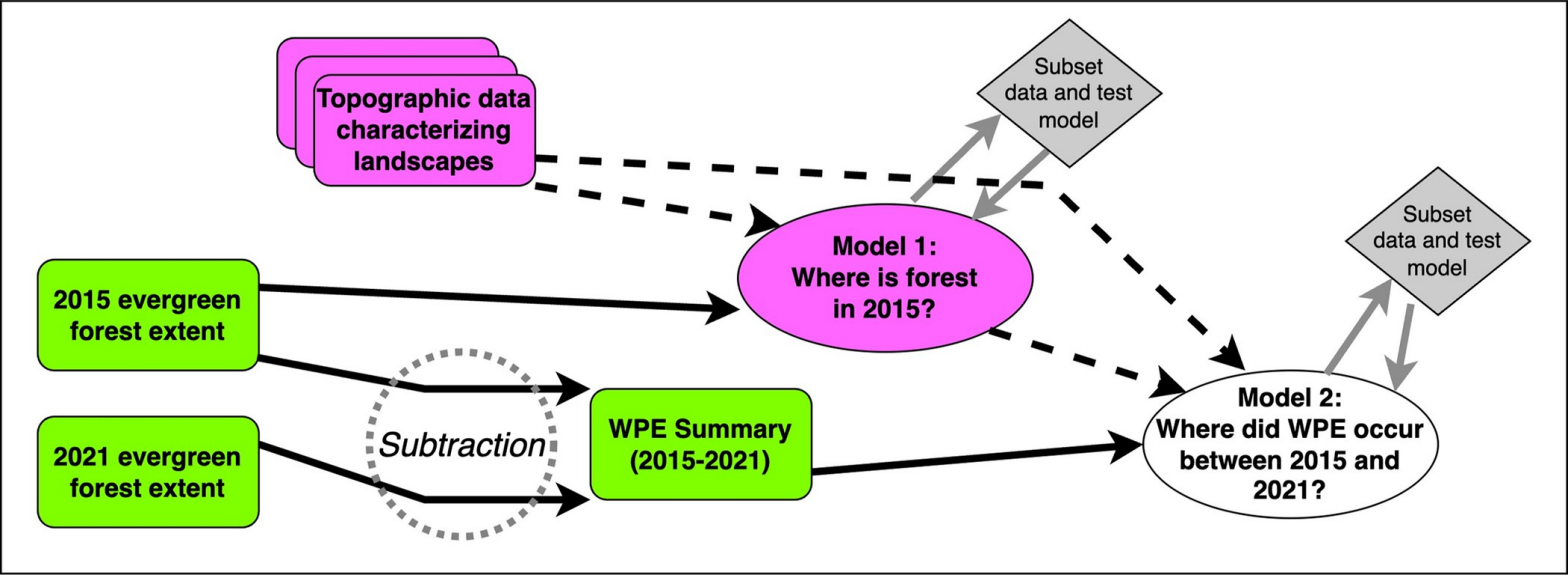
Summary of the flow of information in the analyses reported in this paper. Raster data layers that supply occurrence information (for forest presence or for woody plant encroachment) are shown in green, whereas data layers used as environmental variables are shown in purple. Gray shapes indicate phases of model testing.

The work reported in this paper depends on two major data streams, as well as complex maximum-entropy-based models. The general workflow of the analysis is depicted in Fig 1. Derivation of each of the inputs and development and evaluation of the predictive models are described in the paragraphs that follow.

In all cases, we trained models in one Kansas county (Wabaunsee County, 2057 km^2^), and, once candidate models were calibrated and best models selected, we transferred them across the 8 neighboring counties, where we tested their predictions across nearby, but independent landscapes. As such, this study is cast across a broad area of north-central Kansas, covering much of the northern extent of the tallgrass prairie biome in the state. The region currently holds a mosaic of farmland and rangeland, interspersed with extents of tallgrass prairie in various states of degradation.

### Evergreen woody plant distributions in 2015 and 2021

Identifying and mapping evergreen forests in arid and semi-arid grassland regions over years is the basis for understanding WPE. We use knowledge-based classification methods, microwave images (PALSAR-2), and optical images (Landsat) to generate annual maps of forest and evergreen forests.

The backscatter signal of L-band PALSAR-2 exhibits higher values in forest areas than other land cover types (e.g., grasslands, croplands, water bodies, and bare soil) given its capacity to penetrate tree leaf canopies and interact with tree branches and trunks. Complex textured surfaces such as rocky lands and built-up areas have high backscatter signal, leading to commission errors (i.e., false predictions of presence) in forest signature analysis [18], but these land cover types have no or little vegetation and thus can be effectively identified by low Normalized Difference Vegetation Index (NDVI) values derived from surface reflectance of optical images. Based on these spectral and microwave features, we have developed a forest-cover mapping approach that integrates PALSAR/PALSAR-2 data with optical images (e.g., MODIS, Landsat); the approach has been evaluated in various regions, including monsoon Asia [19], China [20], South America [18], and Oklahoma [21]. These publications used forest definitions from the Food and Agricultural Organization (FAO) Global Forest Resources Assessment [22]. The same methods were used in this study to identify forest and evergreen forest in Kansas.

First, we generated annual maps of forest. Both structure/biomass-sensitive data (HH, HV, HH-HV, and HH/HV) from PALSAR-2 images and vegetation coverage-sensitive data (NDVI_max_) from Landsat 7, 8, and 9 images were used to determine decision rules for forests. Forest pixels are identified by defining specific pixel value ranges: -19 ≤ HV ≤ -7.5, 0 ≤ HH-HV ≤ 9.5, and 0.2 ≤ HH/HV ≤ 0.95. Since forests typically have a higher leaf area index (LAI) than rocky lands, barren lands, and built-up surfaces, which have no or little green vegetation throughout the year, we also applied NDVI_max_ > 0.7 to extract forest pixels, thereby eliminating commission errors in PALSAR-2-based forest maps [23]. In this process, the 25-m PALSAR-2-based forest maps were resampled automatically to 30-m spatial resolution to match Landsat images via the Google Earth Engine (GEE) platform. The resulting forest maps of Kansas in 2015 and 2021 were thus produced by combining PALSAR-2-based forest maps and Landsat-based NDVI_max_ layers.

Second, we identified evergreen forests in the annual forest maps. Evergreen woody plants retain green leaves throughout the year. The differences in leaf phenology between evergreen forests and deciduous forest types can be captured using time series satellite-based vegetation indices (e.g., NDVI, EVI and LSWI) [24]. Seasonal characteristics of NDVI were analyzed based on exploratory analyses of time series imagery for evergreen forest and other forest types in Oklahoma, and it was found that an average NDVI value of 0.4 during winter months (December, January, February) was particularly effective in distinguishing between evergreen forest and other forest types [25]. Considering the similarity of evergreen forests in Oklahoma and Kansas, we applied this threshold (NDVI_mean_ > 0.4) to identify evergreen forest pixels in the forest maps, and refined the annual forest maps into annual maps of forest and evergreen forest across Kansas for 2015 and 2021. We then assumed that evergreen forest pixels in the arid and semi-arid grassland regions appearing in later years and absent in earlier years represent WPE pixels.

### Occurrence data for 2015 red cedar distributions and WPE

From the evergreen forest maps for 2015 and 2021 described above, we derived sets of occurrence data for incorporation into the process of developing ecological niche models. We opted to use random points to represent most faithfully the distribution and extent of evergreen forest patches across the region.

In response to the challenge of deriving models to characterize the distribution of eastern red cedar or juniper in 2015, we created point occurrence datasets for evergreen forest distributions in that year. Specifically, for model calibration and for selection of best models for further exploration, we converted the raster-format 2015 evergreen forest maps to vector format and created 9200 random points within those forested areas across Wabaunsee County (“training data”) for use in model calibration and selection. For the broader area, comprising the 8 counties surrounding Wabaunsee County (i.e., Riley, Pottawatomie, Jackson, Shawnee, Osage, Lyon, Morris, and Geary counties) to provide independent tests of model predictions (“testing data”), conversion to vector format was not possible, so we cast 10,000 random points across the 8-county region, and filtered for those points that fell in forested areas. This set of steps resulted in 9200 unique occurrence points for model training, and 359 unique occurrence points for model testing for the first set of models to be developed in this project.

Second, to respond to the challenge of deriving models that can identify areas where WPE occurred between 2015 and 2021 without any information about the 2021 distribution (i.e., a “hindcasting” analog of prediction), we overlaid 2021 and 2015 evergreen forest cover maps, and focused on areas not holding evergreen forest in 2015 but holding evergreen forest in 2021. Again, for model calibration and selection, we cast 100,000 random points across the entire 9-county study area, from which we filtered the random points to retain only sites that, in the 30-meter resolution forest-cover maps described above, represented WPE areas between 2015 and 2021. This set of steps resulted in 4900 unique occurrence points from Wabaunsee County for model training, and 3938 unique occurrence points for model testing across the surrounding 8 counties for the first set of models to be developed in this project.

### Environmental data

We developed a rich set of raster data layers that summarized dimensions of the landscape that may be relevant to driving either the 2015 distribution of red cedars across central Kansas, or the further encroachment of red cedars between 2015 and 2021. Development of environmental coverages that depend on distances across the 9-county area buffered by 25 km, to avoid any potential problems deriving from edge effects, and to remove negative effects of any undefined peripheral pixels that might otherwise fall in the area of interest.

Given the small spatial extent of our analyses in this paper, we have focused on topographic, landscape-scale features that likely shape local distributions of plant communities; we reviewed other relevant dimensions (e.g., precipitation, vapor pressure deficit, soil characteristics), and found that available datasets were overly coarse, and without dramatic variation across 1–9 Kansas counties, to make them useful in our models. From the GMTED 7.5” mean digital elevation model [DEM, tile 30N120W; 26], we derived (1) elevation (directly from the DEM layer), as well as (2) slope (in degrees), (3) aspect (in 0–360 degrees of orientation), and (4) ruggedness (which calculates the average of the differences between the central pixel and each of the 8 surrounding pixels), all using the Raster Terrain Analysis tool of Raster Analysis functions within QGIS.

To assess (5) distance to creeks and rivers, we assembled two vector-format datasets describing the distribution of creeks and rivers across Kansas [27]. We had to fix a small number of invalid geometry features, using tools in QGIS, and then used the Geoprocessing / Dissolve tool to reduce each dataset to a single feature. Because QGIS tools could not convert these datasets from vector to binary raster format, we performed this single step in ArcGIS. We then, in QGIS, combined the binary creek and river via a raster query, assigning a value of 1 to all pixels in which creeks + rivers > 0, and a value of 0 to all other pixels. We then used the Proximity Distance Raster tool to create a raster file that summarized geographic distance from every pixel to a pixel that falls on either a creek or a river.

Finally, for the second modeling challenge (i.e., anticipating the geographic potential for WPE between 2015 and 2021), we included two further environmental datasets. The first of these datasets was (6) the suitability layer that was derived for 2015 evergreen forests (see above); we used the median output of the first modeling exercise, which approximated suitability for red cedar, at least on the landscapes in the study region in 2015. The second was a raster summary of (7) distance to 2015 evergreen forest extent: we used the Proximity Distance Raster tool in QGIS again to create a raster file that summarized geographic distance from every pixel to a pixel that held evergreen forest in 2015. Pixels holding evergreen forest in 2015 were assigned values of zero in this step.

### Correlative model development and testing

In each of the modeling challenges described above (2015 evergreen forest distribution, WPE during 2015–2021), we took advantage of a flexible platform for maximum-entropy modeling in R, implemented in the kuenm package [28] that has seen massive use in the distributional ecology community. We used an implementation that explores many candidate models with different parameter values for (1) feature types (i.e., the shape of the response curve of dependent to independent variables), (2) regularlization parameter (i.e., the degree of detail in the fit of the response curve to the available information about presences and absences), and (3) the environmental parameters used to define the environmental space. In each case, we assessed candidate models representing all possible combinations of all of the 31 possible combinations of feature types (linear, quadratic, product, threshold, hinge), 6 regularization parameter values (0.3, 0.5, 0.75, 1, 3, 5), and all combinations of the the 5–7 environmental variables. As such, we expolored total numbers of candidate models of 4836 and 22,320, in the two models, respectively.

We chose best models from among the candidate models via an ordered, three-step process implemented in kuenm. Specifically, we first filtered out candidate models that were unable to make statistically significant predictions of a random 50% of available occurrence data across the training region, as evaluated using the partial receiver operating characteristic approach [E = 10%, 500 replicates; 29]. Second, we filtered out models failing to meet a criterion of predictive performance, specifically in omitting ≥10% of the same testing occurrence dataset. Finally, we used the Akaike Information Criterion (AICc) approximation of Muscarella, Galante [30] to assess model fit and complexity (although the assessment of model fit remains somewhat incomplete), retaining as final “best” models those that are ≤2 AICc units away from the minimum across all models passing the first two filters.

**Fig 2:**
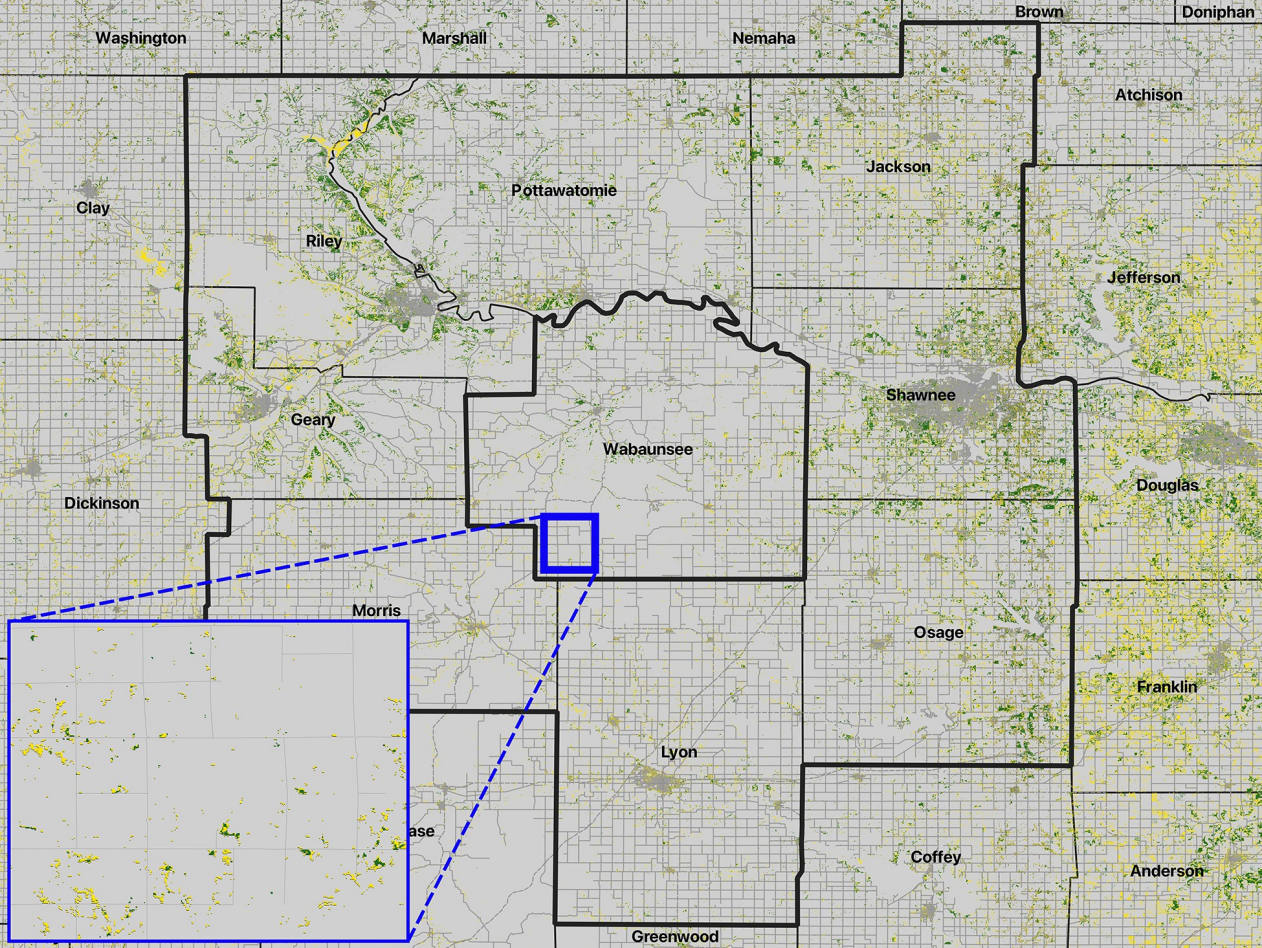
Map showing the study area, and the areas over which 249 models were calibrated and evaluated, in central Kansas. Models were calibrated across Wabaunsee County, in the center of the map, and evaluated in the 8 surrounding counties (outlined in bold black lines. The inset (blue outline) shows a detailed view of a small part of southwestern Wabaunsee County: Yellow areas were forested in 2015, whereas green areas represent areas that became forested between 2015 and 2021.

As a more rigorous, and more fully independent model evaluation test of model predictions, we transferred the “best” models for each of the predictive modeling challenges to the 8 counties surrounding Wabaunsee County (Fig 2) with no extrapolation permitted, thus truncating predictions across the 8 counties at the extreme values of environmental layers as represented within Wabaunsee County. We derived occurrence data for the 2015 extent of evergreen forest and for WPE during 2015–2021 by the approaches described above. We used the “final model evaluation” functions of kuenm to apply a threshold-independent partial ROC test (again E = 10%, 500 replicates) to evaluate whether the model was able to anticipate the phenomenon in question (i.e., 2015 evergreen forest distribution, spatial extent of WPE in 2015–2021) better than a random prediction. This test of predictions across distinct (though adjoining) areas offers a model evaluation that is more rigorous than testing with a subset of the presence data available for the training region [16]. All areal calculations are presented based on analyses of raster-format data in a U.S. National Atlas Equal Area projection (European Petroleum Survey Group projection code 2163).

## Results

Across the extent of Wabaunsee County, evergreen forest (essentially all representing coverage by eastern red cedar) covered an area of 23.6 km^2^ (1.15% of the county) in 2015, and 53.1 km^2^ (2.58%) in 2021. Proportional extent of evergreen forest was greater in the eight surrounding countries, over which our models were evaluated, with 3.53% and 6.46% evergreen forest in 2015 and 2021, respectively. The analyses presented below are based on occurrence points sampled at random from the 2015 extent, and from the areas of WPE between 2015 and 2021.

### 2015 evergreen forest distribution

Of 4836 candidate models that were tested in this analysis, the vast majority (4822) resulted as statistically significant in the partial ROC tests (*P* < 0.05). Of those significant models, however, only 528 also met the omission rate criterion. Finally, of the 528 significant and well-performing models, two were within 2 AICc units of the minimum AICc value (AICc = 194538.2). Those two “best” models were both based on environmental datasets including only elevation and distance to creek, with a regularization parameter value of 0.75, and either threshold features only or threshold and quadratic features. Response curves (see Supplementary Materials) show the direction and shape of these relationships.

**Fig 3:**
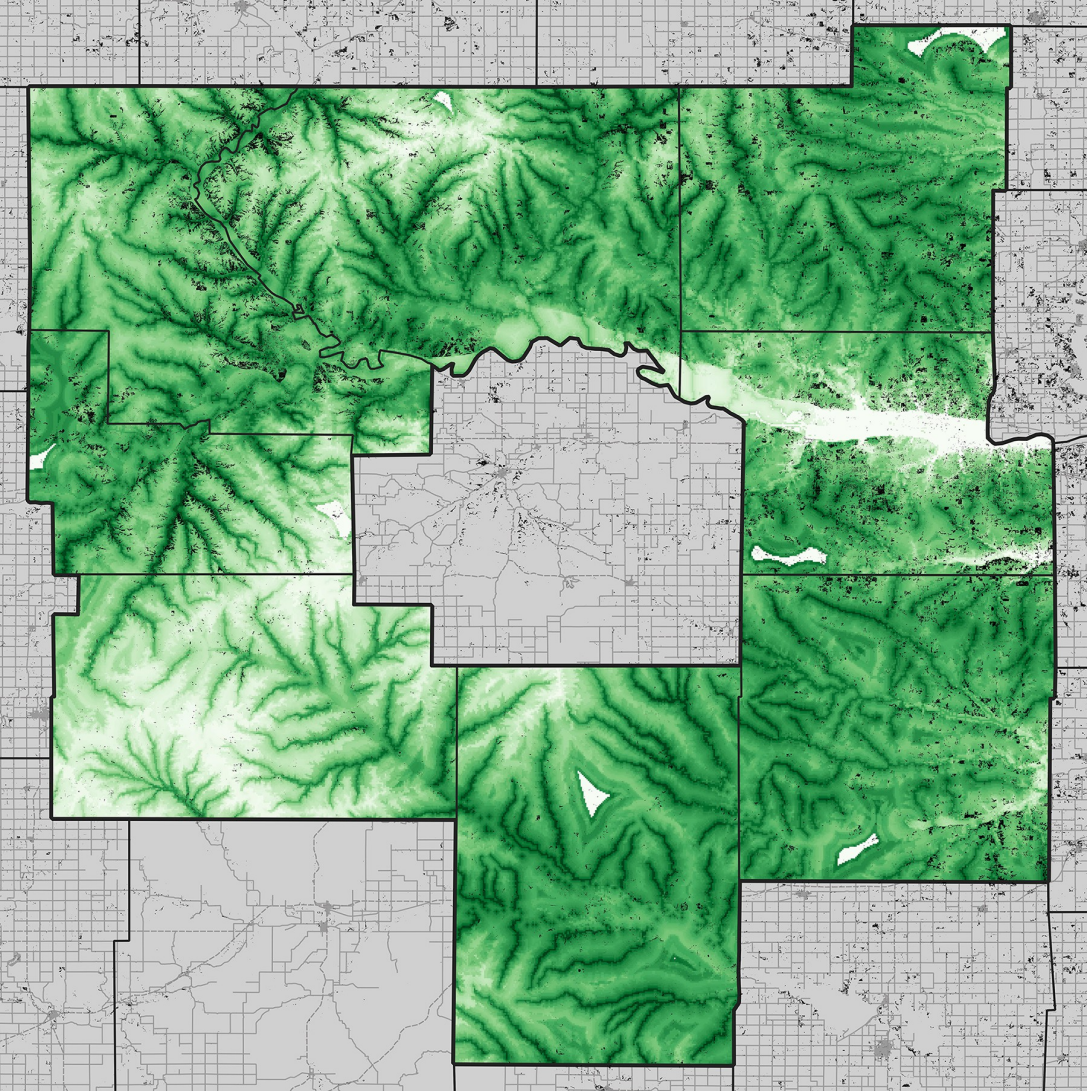
Summary of calibration phase of development of models of 2015 evergreen forest extent across Wabaunsee County. (top left) Distribution of evergreen forest in 2015 and woody plant encroachment during 2015–2021, and (top right) model output (light green = low suitability, dark green = high suitability). The bottom two panels show the two environmental variables that were included in the “best” models that were detected and explored in this study, each shown with lighter colors indicating higher values.

**Fig 4:**
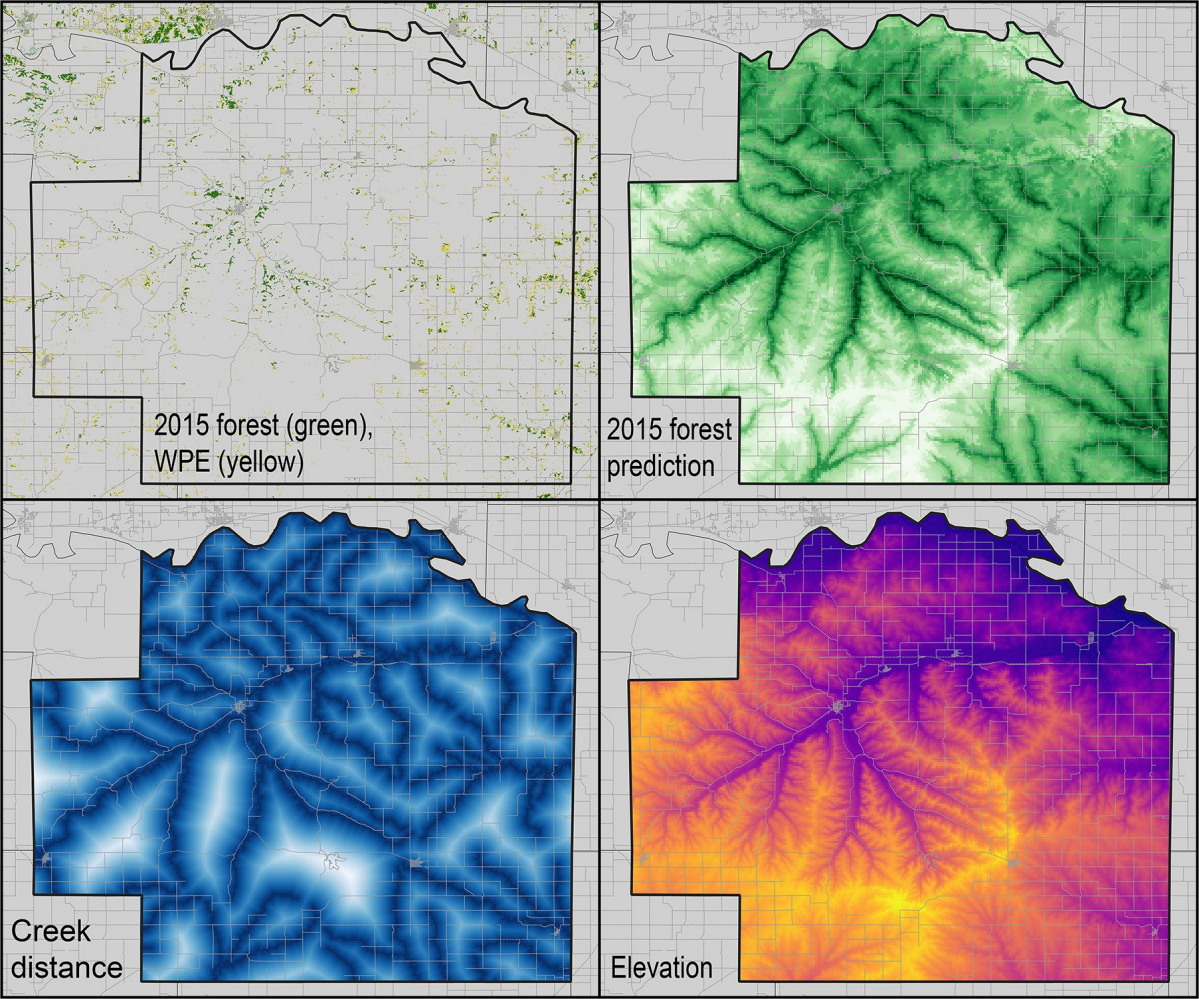
Test of the predictions of 2015 evergreen forest extent in the 8-county test area in central Kansas. Model predictions range from low suitability (white) to high suitability (dark green); actual 2015 evergreen forest extent in the region is indicated by black specks. Small gray areas within the 8-county test area are those in which the model transfers from the calibration region to the test area were extrapolative, and so no predictions were made.

The 2015 evergreen forest model response curves showed maximum suitability at low and high values of creek distance, with an intermediate minimum, and maximum suitability at intermediate elevations. Looking at the model predictions on a map (Fig 3), the relationship to elevation may be somewhat misleading, in that the lowest elevations are along the Kansas River, which is heavily wooded with non-evergreen forest types. As such, the true relationship is probably one that is simply of lower suitability at higher elevations. Transferring this model across the 8-county test area (Fig 4), the coincidence between the actual 2015 distribution of evergreen forest and the model predictions is close, and indeed results as statistically significantly better than random predictions (*P* < 0.05).

### Woody plant encroachment during 2015–2021

**Fig 5:**
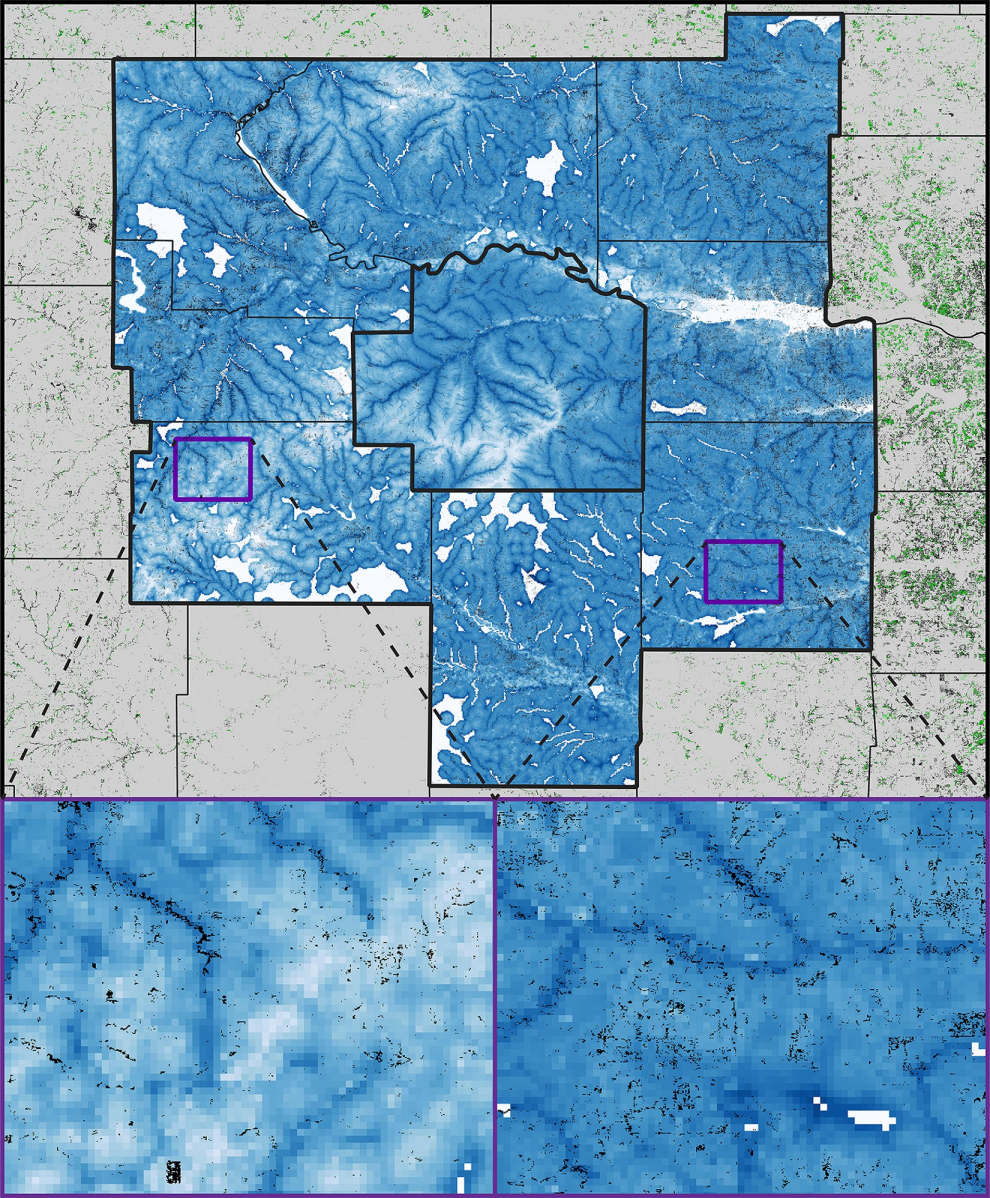
Summary of model results for woody plant encroachment (WPE) in terms of increased extent of evergreen forest in central Kansas between 2015 and 2021. Top panel shows full extent of the study area, with 2015 evergreen forest extent shown in green, and WPE during 2015–2021 shown in black; both are plotted on top of the model predictions (light blue = unsuitable, dark blue = highly suitable; light gray = no predictions made under extrapolative conditions), which were calibrated in Wabaunsee County (center of map) and evaluated across the 8 surrounding counties. The bottom two panels show “zooms” to two areas in the evaluation area, such that predictions and WPE extent (in black) are independent.

Of 22,320 candidate models that were tested in this analysis, the vast majority (21,895) resulted as statistically significant in the partial ROC tests (*P* < 0.05). Of those significant models, however, only 3471 also met the omission rate criterion. Finally, of the significant and well-performing models, we identified a single model on the basis of having a low value of AICc, and hence low complexity of the model; no other model was within 2 AICc units that value, and thus no other model is considered to be equivalent in terms of complexity). The “best” model was based on a broad suite of environmental datasets including distance to creek, distance to 2015 evergreen forest, ruggedness, slope, and suitability from the 2015 evergreen forest suitability model described above (Fig 5). The “best” model had a regularization parameter value of 0.5, and was based on linear, product, and hinge feature types. Response curves (see Supplementary Materials) show the direction and shape of these relationships.

The 2015 evergreen forest suitability surface had a positive relationship; the remaining variables had rather more subtle effects on the response variable. Transferring this model across the 8-county test area (Fig 5), the coincidence between the actual pattern of WPE across the region and the model predictions is close, and indeed resulted as statistically significantly better than random predictions (*P* < 0.05).

## Discussion

**Fig 6:**
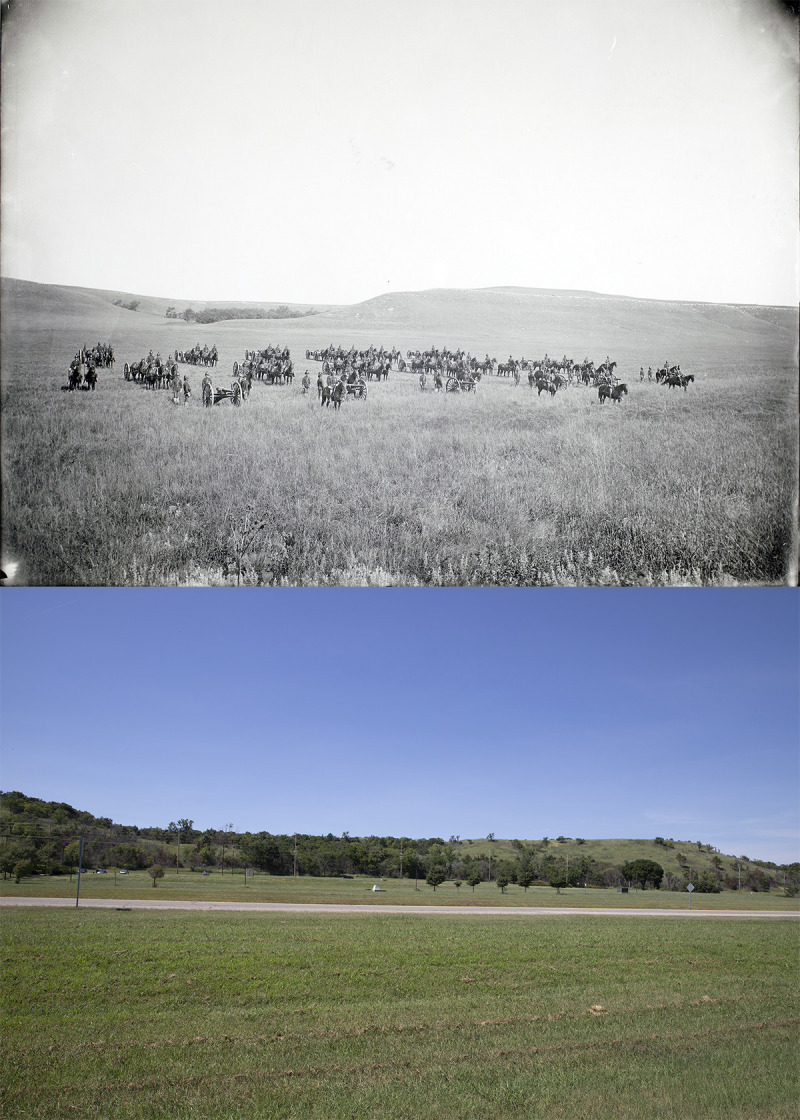
Comparison of old and new views on Ft. Riley, Geary County, Kansas (Kansas Rephotography ID number 2226). Top photo shows a view from 1899, showing military maneuvers on Pawnee Flats, on Ft. Riley (photo courtesy of the University of Kansas Libraries). Bottom photo was taken from 39.0901°N, 96.7538°W in 2023. Note the original restriction of forested areas to the valley, but the extension of the treed areas in the valleys and onto some of the higher areas in the recent photo. Top portion of figure modified from an image provided at https://digital.lib.ku.edu/ku-pennell/842, under a CC BY license, with permission from University of Kansas, original copyright 1899.

This study has explored the possibility of landscape characteristics being used to develop predictive models of the geographic potential for woody plant encroachment into grassland ecosystems. Our approach is based on concepts and methodological frameworks drawn from the field of distributional ecology [16]. Although the analogy with the fundamental ecological niche of an individual species is not complete in this case, and the model being fit is clearly not a full, universal niche response. Nonetheless, for prairie landscapes in central Kansas, our analyses indicate considerable potential utility in anticipating both evergreen forest distributions and the areas into which they expand over time—a photographic example of this process in Wabaunsee County, Kansas, where our models were calibrated, is shown in Fig 6.

### Predictions across central Kansas

The models developed and explored herein are applicable only on local scales and extents, and are clearly not generally applicable, even at the level of the Great Plains region or the full state of Kansas. That is, they are defined in terms that are locally meaningful, such as a particular elevational range, which is certain not to be the correct or appropriate elevational range in other regions or on other landscapes. As such, although this study cannot be interpreted as a step towards a universal predictive model of WPE, even across only the tallgrass prairie biome, it serves to illustrate that WPE is a phenomenon that has predictable dimensions across landscapes.

The predictions developed in this study for central Kansas are, in both cases, statistically significantly better than those of many replicate null models. Although statistically significant, our model predictions are nonetheless not perfect and absolute. For example, several clear exceptions can be noted in the WPE predictions shown in Fig 5, which are likely a function of human planting of the same species (or possibly planting other evergreen tree species). As such, the models appear to be capturing the essence of the landscape conditions under which 2015 evergreen forest was distributed and under which WPE occurred between 2015 and 2021, yet the relationship is certainly not absolute.

### Conclusions: WPE processes and predictions

WPE is a phenomenon that is decidedly complex and multifactorial, with causes including fire suppression, changing grazing regimes, shifting human behavior, and others [9,31]. Considerable effort has been invested in developing remote-sensing approaches to characterizing WPE processes retrospectively [8,25,32]. However, less attention has been paid to the more exploratory challenge of anticipating areas with greater or lesser potential for further WPE [33,34]. A notable exception is the work of Donovan, Burnett [35], who found that WPE from windbreaks was most likely along roadways, and less along other features, suggesting a predictive local nature to WPE, albeit in a different landscape context than that of this study.

This study offers a further exploration of the predictive nature of WPE phenomena. That is, we have taken a suite of methods and tools from the world of biogeography and ecology [16], specifically tools for developing what are termed species distribution models or ecological niche models by relating known occurrences of a species or phenomenon to raster data layers that summarize relevant dimensions of the environment. The output is a map that summarizes similarity in environmental dimensions between all of the landscape and the sites where a species or a phenomenon exists or occurs. As such, this study explores methodologies that are quite distinct from those used by Donovan, Burnett [35], yet the results are complementary: WPE is a phenomenon that involves expansion of local ranges of tree species (in our case, red cedar) in consistent portions of the environmental space that characterizes the overall landscape. Future such analyses that extend over broader areas would need to integrate the landscape-scale factors assessed in this paper with climate- and soil-related data dimensions that are important across larger-scale gradients. In this sense, we offer a contribution that augments the discussion to date about predictive and prospective evaluations of WPE dynamics.

## Supporting information

S1 File Details of modeled responses to different environmental variables in the different “best” models for the evergreen forest model and in the woody plant encroachment model. (DOCX)
