# Supplementary material for: Correlative ecological niche model applications to predicting landscape-scale woody plant encroachment in Kansas tallgrass prairie systems
Source: PLoS One. 2024 Jun 13;19(6):e0305168. doi: 10.1371/journal.pone.0305168 (PMC11175484; doi:10.1371/journal.pone.0305168)

**2015 Evergreen Forest Distribution Model**

Model 1: Regularization parameter value 0.75, quadratic and threshold response types


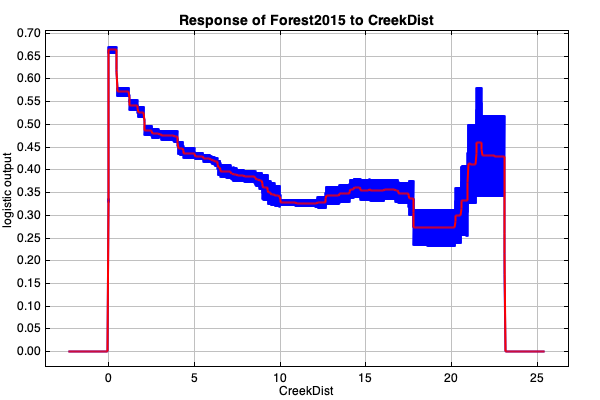


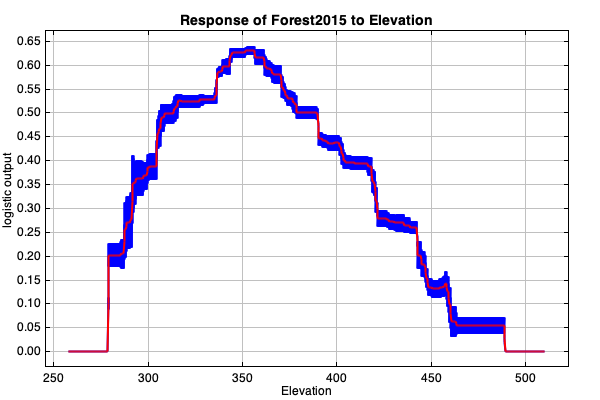


Model 2: Regularization parameter value 0.75, threshold response types


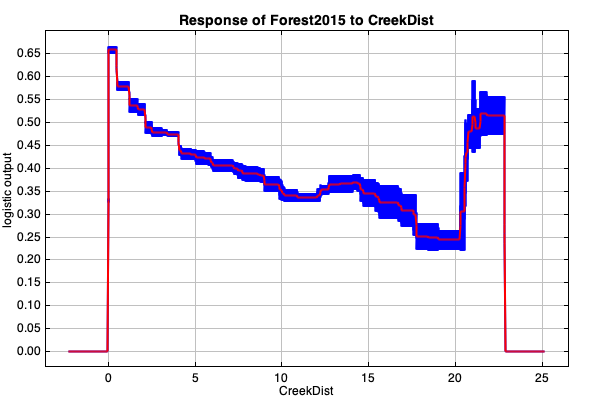


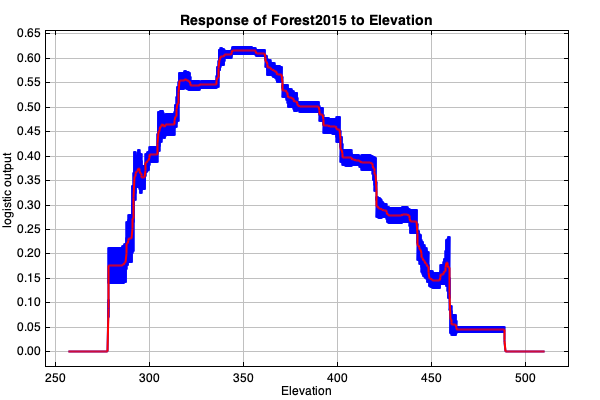


**2015-2021 Woody Plant Encroachment Model**

Model: Regularization parameter value 0.50, response types linear, product, and hinge


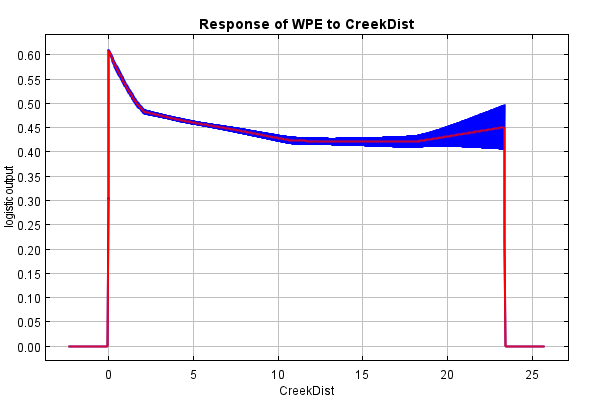


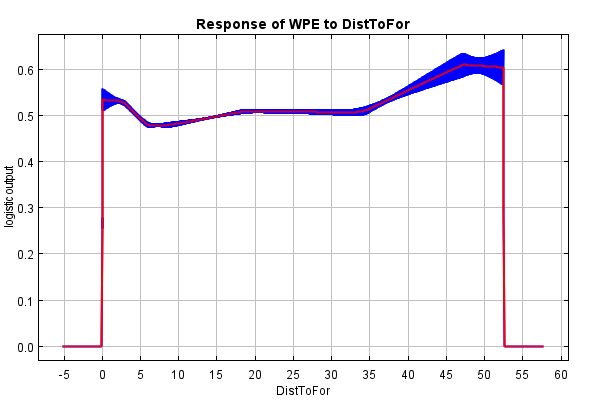


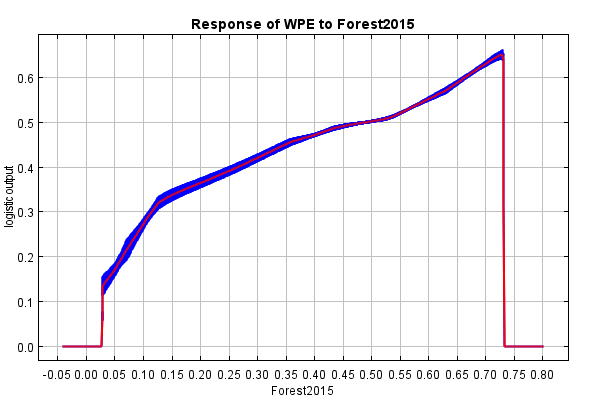


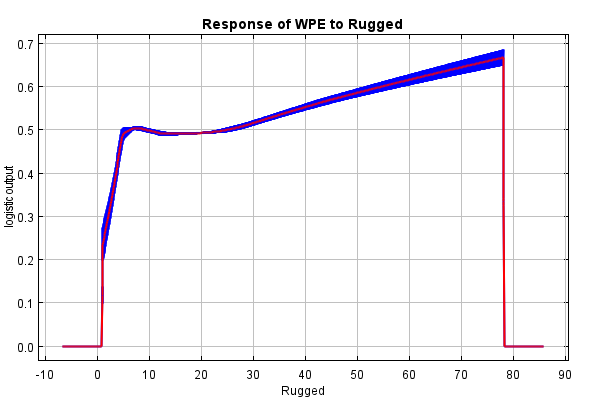


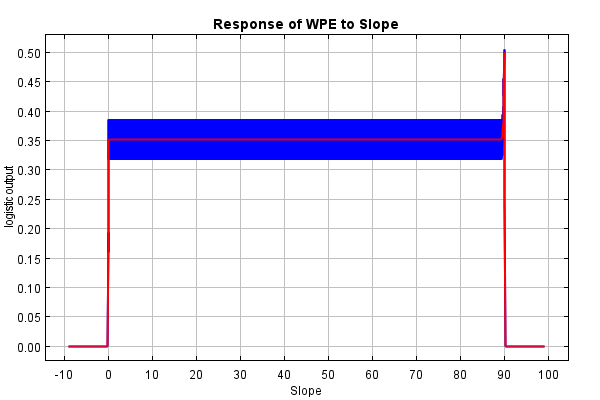

Supplement: S1 File — (DOCX) [file pone.0305168.s001.docx]
